# Functional Polymers in Sensors and Actuators: Fabrication and Analysis

**DOI:** 10.3390/polym12071569

**Published:** 2020-07-15

**Authors:** Akif Kaynak, Ali Zolfagharian

**Affiliations:** School of Engineering, Deakin University, Geelong, Victoria 3216, Australia

**Keywords:** functional polymers, sensors, actuators, 3D printing, 4D printing

Recent advances in fabrication techniques have enabled the production of different types of polymer sensors and actuators that can be utilized in a wide range of applications, such as soft robotics, biomedical, smart textiles and energy harvesting. This Special Issue focuses on the recent advancements in the modeling and analysis of functional polymer systems.

The first paper published in the issue presents the work of Yu and colleagues in Shandong University, China, in which they synthesized hydrogel materials that could respond to the surrounding environment by a color change inspired by nature [1]. The researchers have presented an efficient method to improve the photonic sensing properties of polymeric gels by using non-close-packed monolayer colloidal crystals as the template. The authors developed an ultrathin photonic polymer gel film which exhibited significant improvement in responsiveness and linearity towards pH sensing compared to those prepared earlier, achieving fast visualized monitoring of pH changes with excellent cyclic stability and a small hysteresis loop.

In the second article, a collaborative research team, including the University of Tartu, Estonia and Ton Duc Thang University, Vietnam, developed software for driving and measuring ionic electroactive material-based systems [2]. A set of functions, hardware drivers, and measurement automation algorithms were developed in the National Instruments LabVIEW 2015 system to control synchronized isotonic (displacement) and isometric (force) measurements over a single compact graphical user interface called electro-chemo-measurement software (IIECMS). The suitability of the proposed software was successfully tested on the two different materials representing high stress, strain and low strain characteristics.

The Special Issue progresses to the third manuscript with the work of Park and colleagues from Inje University, South Korea, on ionic electroactive polymer actuators (IEPAs) which are interesting for their flexibility, lightweight composition, large displacement, and low-voltage activation [3]. They have developed a graphene oxide–silver nanowire (GO–Ag NW) based IEPA with Triton X-100 nonionic surfactant to transform the PEDOT:PSS capsule into a nanofibril structure. The fabricated actuator in this work showed improvements in stability, electrical conductivity, and driving performance.

In the fourth article, in an international collaboration, Wang from Donghua University and other colleagues from Texas Tech University and California State University, Fullerton, developed a 3D-printed wearable strain sensor with promising conductivity and transparency suitable for healthcare and soft robotics applications [4]. They combined agar and ionic thermo-responsive alginate to improve the shape fidelity of the hydrogel for 3D printing. With the addition of agar, the rheological characteristic of the 3D printing ink was enhanced for precision printing. In addition, alginate was used to improve the mechanical characteristics of the sensor to a level required for the so-called “electronic skin”.

The researchers in Prince of Songkla University presented the fifth article investigating electrostrictive polymers with applications in biomedical sensors, actuators and energy harvesting devices [5]. The authors worked on increasing the dielectric properties and microstructural β-phase in the poly(vinylidene fluoride-hexafluoropropylene) (P(VDF-HFP)) by optimizing electrospinning conditions and thermal compression. The high electrostatic field in the electrospinning process caused orientation polarization, which helped transform the non-polar α-phase to electroactive β-phase in the formed fibers. Additionally, the increase in compression temperature of up to 80 ℃ resulted in an increase in the crystallinity and the dielectric constant. The results showed the efficacy of the proposed method to improve electrostriction behavior based on the dielectric permittivity and interfacial surface charge distributions for applications in actuator devices, textile sensors, and nanogenerators.

The seventh contribution to the Special Issue focused on the bending problem of a piezoelectric cantilever beam via theoretical and experimental methods. Due to the extensive applications of piezoelectric polymers in the design of intelligent structures, including the sensors and actuators, Yang and associates from Chongqing University, proposed a method to deal with the challenge of solving the governing equations of these materials due to the force–electric coupling characteristics [6]. To do so, they derived the theoretical solution of the bending problem of piezoelectric cantilever beams by the multi-parameter perturbation method, which is a general analysis method for solving approximate solutions of non-linear mechanical problems. The solution of their proposed method was successfully validated with the experimental results as well as existing solutions in the literature.

International researchers from Nottingham Trent University, University of Tehran, Deakin University, and University of Glasgow conducted a new level of study in digital fabrication, publishing their findings in harnessing variable bandgap regions by 4D printing via shape-adaptive metastructures [7]. Focusing on how four-dimensional (4D) metastructures could filter acoustics and transform filtering ranges, the authors used fused deposition modeling (FDM) printing with a single printer nozzle to experiment with shape memory polymer (SMP) materials with self-bending features. Additionally, the mechanism for the creation of metastructures capable of manipulating elastic wave propagation to find bandgaps was demonstrated. The authors claim that the state of the art 4D printing unlocks potentials in the design of functional metastructures for a broad range of applications in acoustic and structural engineering, including sound wave filters and waveguides.

The eighth contribution to the issue is a review of polymer-based microelectromechanical systems (MEMS) electromagnetic (EM) actuators and their implementation in the biomedical engineering field written by a national collaboration of Yunas and colleagues among three universities in Malaysia [8]. The study highlighted the recent development of electromagnetically driven microactuators in terms of the materials, mechanism of the mechanical actuation, and the state of the art of the membrane developments for biomedical applications, such as lab-on-chip and drug delivery systems. The authors envisaged that the polymer composites will eliminate the need for a conventional bulky permanent magnet in electromagnetic actuators in the near future.

This issue finalizes with the work of researchers from the School of Engineering at Deakin University on the development of the wearable strain sensors [9]. In this work, an electrically conductive dynamic hydrogel was designed and produced by incorporating ferric ions and tannic acid-coated chitin nanofibers (TA-ChNFs) into the hydrogel network. TA-ChNFs had a reinforcing role as nanofillers and also acted as dynamic cross-linkers, thus imparting an outstanding self-healing ability and high strength to the hydrogel. Moreover, the hydrogel displayed excellent stability with repeatable self-adhesive properties, with the ability to attach to almost any surface. This electroconductive and tough hydrogel with autonomous self-healing and self-recovery properties appeared to be an excellent candidate for wearable strain sensing devices. 
